# Estimating *Ixodes ricinus* densities on the landscape scale

**DOI:** 10.1186/s12942-015-0015-7

**Published:** 2015-08-14

**Authors:** Denise Boehnke, Katharina Brugger, Miriam Pfäffle, Patrick Sebastian, Stefan Norra, Trevor Petney, Rainer Oehme, Nina Littwin, Karin Lebl, Johannes Raith, Melanie Walter, Reiner Gebhardt, Franz Rubel

**Affiliations:** Institute of Geography and Geoecology, Karlsruhe Institute of Technology, Reinhard-Baumeister-Platz 1, 76131 Karlsruhe, Germany; Institute for Veterinary Public Health, University of Veterinary Medicine Vienna, Veterinärplatz 1, 1210 Vienna, Austria; Department of Ecology and Parasitology, Institute of Zoology, Karlsruhe Institute of Technology, Kornblumen Strasse 13, 76131 Karlsruhe, Germany; Baden-Württemberg State Health Office, District Government Stuttgart, Nordbahnhof Strasse 135, 70191 Stuttgart, Germany; Institute of Applied Geosciences, Karlsruhe Institute of Technology, Adenauerring 20, 76131 Karlsruhe, Germany; Institute of Mineralogy, Technische Universität Bergakademie Freiberg, Brennhausgasse 14, 09596 Freiberg, Germany

**Keywords:** Ixodid ticks, Generalized linear model, Population density, Climate, Land cover classification

## Abstract

**Background:**

The study describes the estimation of the spatial distribution of questing nymphal tick densities by investigating *Ixodes ricinus* in Southwest Germany as an example. The production of high-resolution maps of questing tick densities is an important key to quantify the risk of tick-borne diseases. Previous *I. ricinus* maps were based on quantitative as well as semi-quantitative categorisations of the tick density observed at study sites with different vegetation types or indices, all compiled on local scales. Here, a quantitative approach on the landscape scale is introduced.

**Methods:**

During 2 years, 2013 and 2014, host-seeking ticks were collected each month at 25 sampling sites by flagging an area of 100 square meters. All tick stages were identified to species level to select nymphal ticks of *I. ricinus*, which were used to develop and calibrate Poisson regression models. The environmental variables height above sea level, temperature, relative humidity, saturation deficit and land cover classification were used as explanatory variables.

**Results:**

The number of flagged nymphal tick densities range from zero (mountain site) to more than 1,000 nymphs/100 m^2^. Calibrating the Poisson regression models with these nymphal densities results in an explained variance of 72 % and a prediction error of 110 nymphs/100 m^2^ in 2013. Generally, nymphal densities (maximum 374 nymphs/100 m^2^), explained variance (46 %) and prediction error (61 nymphs/100 m^2^) were lower in 2014. The models were used to compile high-resolution maps with 0.5 km^2^ grid size for the study region of the German federal state Baden-Württemberg. The accuracy of the mapped tick densities was investigated by leave-one-out cross-validation resulting in root-mean-square-errors of 227 nymphs/100 m^2^ for 2013 and 104 nymphs/100 m^2^ for 2014.

**Conclusions:**

The methodology introduced here may be applied to further tick species or extended to other study regions. Finally, the study is a first step towards the spatial estimation of tick-borne diseases in Central Europe.

**Electronic supplementary material:**

The online version of this article (doi:10.1186/s12942-015-0015-7) contains supplementary material, which is available to authorized users.

## Background

The study describes the estimation of the spatial distribution of questing tick densities using the example of *Ixodes ricinus* in Southwest Germany. *Ixodes ricinus* is the most well-known and studied European tick species transmitting various arthropod-borne diseases [1, 2]. Suitable habitats are found all over in Germany [3]. The knowledge of the spatial distribution of tick densities is a key factor in quantifying the risk of tick-borne diseases such as tick-borne encephalitis, Lyme borreliosis, anaplasmosis, rickettsiosis, and others.

Previous studies on the spatial distribution of ticks mainly comprise the determination of the presence and absence of single or multiple species. Frequently, these tick data were exclusively published for selected sites, but recently more and more models for spatial interpolation or analysis have been developed. These spatial distribution models, also known as ecological niche models, are generally applied on a continental scale. Therefore, various statistical models to describe species habitats have been developed. An overview on models as well as methodological caveats in the modelling with examples for *I. ricinus* was described by Estrada-Peña and co-authors [4]. Several applications focus on the projection of climate niche models to estimate future scenarios. These studies provide maps of suitability indices and presence/absence maps of both the present and the future distribution of *I. ricinus*, covering Europe and Northern Africa [4, 5].

**Fig. 1:**
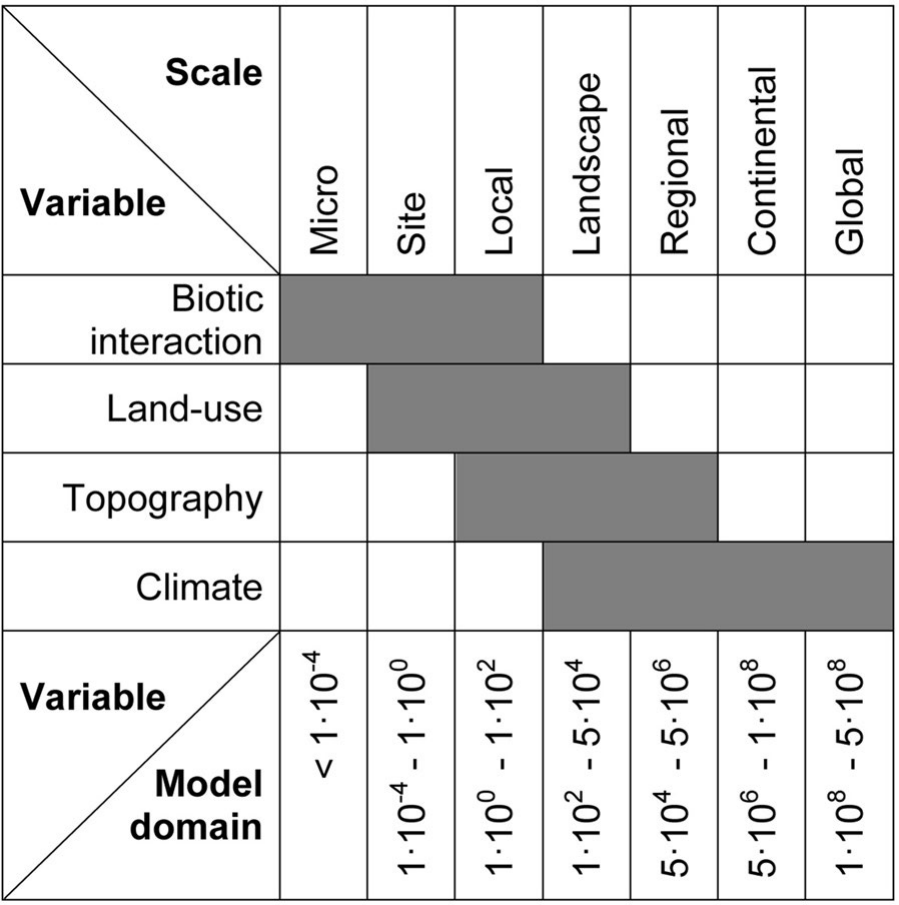
Environmental variables affecting tick densities at different spatial scales. Scales are characterized by the model domain in km^2^, adapted from [13].

Calculations of the spatial distribution of tick densities, however, are rare. The few papers available comprise the mapping of the nymphal density of *I. pacificus* in California using GIS and satellite derived data [6], the mapping of the density of *I. scapularis* in the eastern United States using geo-statistical interpolation [7] and a zero-inflated negative binominal model [8]. In Europe, the spatial distribution of tick densities was exclusively estimated on local scales. Studies comprise the mapping of *I. ricinus* densities at two sites in the Czech Republic [9] and in a German nature reserve [10]. The two latter, however, are simply based on semi-quantitative categories of tick densities classified according to the number of ticks collected at different study sites, which were characterised by typical vegetation cover. No statistical models were developed for these density maps [9, 10]. In a further local scale analysis [11], generalized estimating equations were applied for mapping of *I. ricinus* densities in an Italian nature reserve (model domain: 5 km^2^). Although other statistical models for *I. ricinus* were developed, these were not used to compile density maps [12]. In contrast, the study presented here, is a contribution to the quantitative spatial density estimation of ixodid ticks on the landscape scale (35,750 km^2^). Here, the landscape scale is defined for model domains of 100–50,000 km^2^ (Fig. 1), where the environmental variables land-use, topography and climate are affecting the distribution of ticks. Thus, biotic interactions expressed for example by vector-to-host ratios and other local phenomena were not considered.

A total of 25 sampling sites in Southwest Germany, in the federal state Baden-Württemberg, were selected according to their vegetation cover and tick habitat suitability. *Ixodes ricinus* was flagged over an area of 100 m^2^ each month from March to October for each site. These field data were used to develop a generalised linear model (Poisson regression) describing the total number of nymphal ticks at the different sampling sites, which were collected during 1 year by monthly flagging an area of 100 m^2^. Air temperature, air humidity, height above sea level and land cover classification were used as explanatory variables. These were preselected according to their biological relevance. Air temperature was selected, since thermal conditions determine the rate of tick development from stage to stage and activity patterns, which in turn influence survival success and population density [10, 14, 15]. This, and potentially other synergetic processes, leads to decreasing tick densities with increasing altitude [14]. Ticks and other terrestrial vertebrates must maintain their body water balance to survive. In a subsaturated atmosphere, unavoidable body water losses in ticks occur, e.g., by discharging faeces and urine, by gas exchange, and via the cuticle. Saturation deficit, the difference between the saturation vapour pressure at 100 % relative humidity (at a given temperature) and the given vapour pressure, is to a large extent a measure of the drying power of the atmosphere and as such largely responsible for the rate of body water loss of a given tick in a given situation [16]. Unfed ticks and some engorged ticks are capable of compensating suffered body water losses by active water vapour uptake when the ambient relative humidity surpasses a certain threshold, the so-called critical equilibrium humidity [17]. As a consequence, both saturation deficit and relative humidity must be taken in consideration when investigating the influence of humidity on ticks (Olaf Kahl 2015, personal communication). The habitat type influences not only the microclimatic conditions, but also the host spectrum and abundance. *Ixodes ricinus* prefers woodland habitats such as broadleaved, mixed or coniferous forests [10, 15].

As a result, predictive models were applied to extrapolate the tick densities to the entire region of Baden-Württemberg. A spatial resolution of 30 arc seconds, corresponding to about 0.5 km^2^, was selected to depict maximum features of the tick density taking into account simultaneously the resolution of the climate data. Although the tick density maps were exclusively developed for *I. ricinus*, the method may be applied for other tick species as well.

## Methods

### Study area

**Fig. 2:**
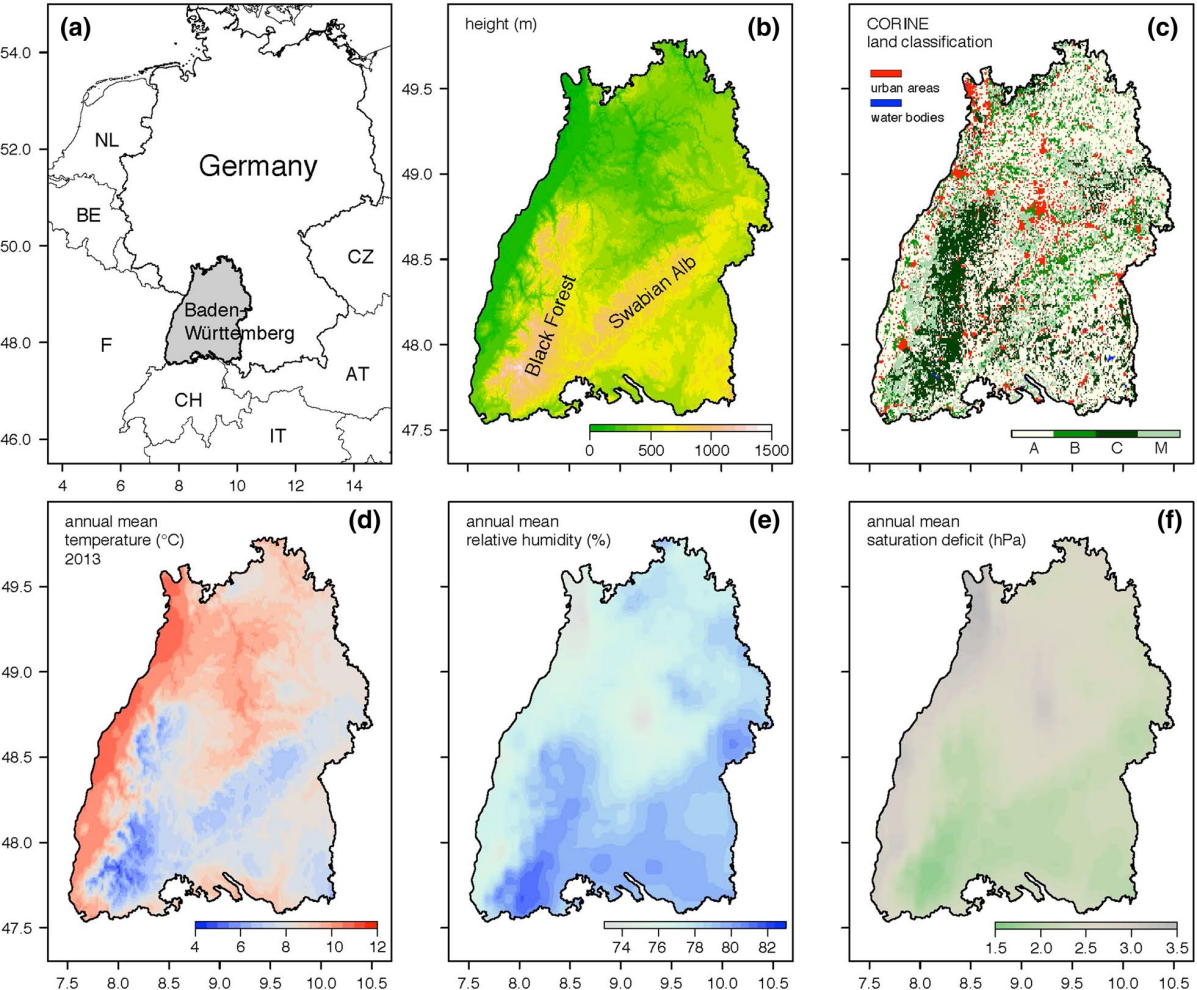
Study area and explanatory variables used to calculate nymphal densities. Study area Baden-Württemberg located in the southwest of Germany (**a**), height above sea level in meters (**b**), CORINE land cover classification (**c**), temperature in °C (**d**), relative humidity in % (**e**) and saturation deficit in hPa (**f**).

The model domain covers the entire region of the German federal state Baden-Württemberg with an area of 35,750 km^2^. It is located in the southwest of Germany (Fig. 2a) and has a wide ecological variability with low lands and mountains up to 1,500 m height above sea level (Fig. 2b). The historical natural coverage was woodland with European beech (*Fagus sylvatica*) as the main tree species. Today the natural landscape has been widely replaced by areas of anthropogenic utilization. The main tree species is the economically important European spruce (*Picea abies*) with 38 % followed by European beech with 21 % of the total forest area. Following the well-known Köppen-Geiger climate classification [18], the climate conditions in Baden-Württemberg as well as in the entire region of Germany were characterized by Cfb climate (C = warm temperate climate, f = fully humid, b = warm summers). Even under climate change conditions the Cfb climate in Baden-Württemberg will be preserved [19]. Total annual precipitation varies between 600 and 2,000 mm.

### Tick sampling and in situ measurements

From March to October (period 2013–2014) host-seeking ticks were collected at 25 sites (Table 1) by dragging a 1 m^2^ cotton flag over 100 m^2^ of woodland scrub and litter. The cloth was checked visually every 10 m^2^ to remove adult and nymphal ticks, which were collected in separate vials for every drag. The standardised sampling took place every month on dry days (no rain) at comparable daytime. All tick stages were identified to species level [20–22], but only nymphal ticks of *I. ricinus* were used in this study. Questing tick density was calculated as the total number of nymphs monthly collected per 100 m^2^ during 1 year, following Schulz and co-authors [15]. At each sampling site a weather station was operated throughout the year. From these continuous measurements, mean annual values of air temperature and relative humidity were calculated. The saturation deficit was estimated by applying the Magnus formula [23], which provides a functional relationship between saturation vapour pressure, temperature and relative humidity. Main vegetation cover was determined according to the *coordination of information on the environment* (CORINE) land classification [24]. Finally, geographical coordinates and the heights above sea level were documented (Table 1).

**Table 1:** Specification of the 25 sampling sites in Baden-Württemberg, Germany

No	Acronym	Site name	Lon	Lat	H_S_	H_G_
1	AH	Allerheiligen	8.1814	48.5337	773	766
2	AL	Altenheim	7.7796	48.4693	147	147
3	AW	Auwald	8.3765	49.1338	111	98
4	BT	Botnang	9.1300	48.7877	349	359
5	CW	Calw	8.8278	48.7419	532	452
6	DS	Drackenstein	9.6562	48.5566	755	730
7	EP	Eppingen	8.9260	49.1107	268	265
8	FB	Feldberg	8.0382	47.8623	1,287	1,284
9	FN	Friedrichshafen	9.5121	47.6515	425	420
10	FR	Freiburg	7.8816	47.9962	383	405
11	GH	Gosheim	8.7576	48.0999	992	882
12	HQ	Hedwigsquelle	8.4356	48.9545	215	242
13	HW	Hardtwald	8.4798	49.1340	117	108
14	KT	Kirchheim Teck	9.4284	48.6286	338	353
15	MB	Michaelsberg	8.5726	49.0882	253	243
16	NA	Neckaraue	9.2060	48.9862	202	185
17	PH	Parzelle Hohenheim	9.0898	48.6801	472	449
18	PK	Parzelle Karlsruhe	8.4257	49.0280	126	123
19	RF	Rosenfeld	8.7241	48.3219	511	502
20	ST	Staffort	8.5115	49.0763	122	111
21	SW	Gaistal Schwarzwald	8.4425	48.7740	610	609
22	VS	Villingen-Schwenningen	8.5666	48.0757	700	693
23	WP	Wippingen	9.8492	48.4153	659	498
24	WR	Wüstenrot	9.4344	49.0994	505	510
25	WU	Wurzacher Ried	9.9253	47.9390	665	653

Site specific parameters comprising the acronym, the site name, the geographical longitude (lon) and latitude (lat) in decimal degrees as well as the height in meters above sea level at the sampling site (H_S_) and of the corresponding grid (H_G_). Note that H_G_ was manually adjusted for CW, GH and WB by choosing the neighbouring grid box to avoid unrealistic deviations between H_S_ and H_G_.

### Gridded environmental data

The gridded data are variables available on a high-resolution raster covering the entire study area. They were used to predict the spatial distribution of the tick density. Exclusively variables with biological significance for the life cycle of ticks were used [15, 25–28]. Figure 2 depicts these explanatory variables comprising the height above sea level, the CORINE land cover classification as well as the air temperature, relative humidity and saturation deficit.

Gridded temperature fields for Germany were provided by the climate data centre of the German weather service [29]. This dataset covers the period from 1881 up to present with a temporal resolution of 1 day and a spatial resolution of 1 km. The mean annual temperature for the years 2013 (Fig. 2d) and 2014 (not shown) were calculated and applied. As humidity measures are not included in this dataset, they were taken from the HYRAS dataset [30]. The HYRAS dataset comprises gridded data with a temporal resolution of 1 day and a spatial resolution of 5 km for the period 1951–2006. As this dataset does not cover the period 2013–2014, climate means (period 1976–2005) of the relative humidity (Fig. 2e) and the saturation deficit (Fig. 2f) were used. Additionally, the vegetation cover was classified using the CORINE land cover dataset [24]. It comprises a total of 44 land cover classes derived from satellite imagery. The recent version of the CORINE data was issued 2013 providing a spatial resolution of 3 arc seconds (<100 m). CORINE level I is divided into five classes: *artificial surfaces*, *agricultural areas*, *forest and semi natural areas*, *wetlands* and *water bodies*. For this study, agricultural areas were denoted as A. Further, from the forest and semi natural areas only the CORINE level III subclasses broad-leafed forest (B), coniferous forest (C), and mixed forest (M) were used (Fig. 2c). No tick densities were estimated for urban areas and water bodies. Thus, the n = 25 sampling sites were divided into 4 land cover categories. Ideal for model calibration the land cover classes were uniformly distributed over the sampling sites, which is reasonably met for 7 sampling sites characterised by class A, 7 by class B, 6 by class C, and 5 by class M (Table 2). Note that the largest and enclosed woodland area is located in the mountainous region of the Black Forest (Fig. 2b). All other regions are characterised by a fragmented mixture of agricultural areas, forests and residential areas.

**Table 2:** Tick densities and environmental variables for the 25 sampling sites

No	Site	N_2013_	N_2014_	T_2013_	T_2014_	RH	SD	LC
1	AH	3	4	7.3	9.0	78.0	2.5	C
2	AL	263	374	10.6	12.2	74.9	3.3	B
3	AW	226	163	10.7	12.4	75.0	3.3	A
4	BT	1,066	169	9.7	11.3	74.8	3.0	B
5	CW	142	68	8.3	10.0	76.5	2.6	M
6	DS	63	82	7.3	8.9	77.0	2.5	A
7	EP	158	44	9.6	11.2	76.7	2.9	B
8	FB	0	0	5.8	7.4	79.9	1.7	C
9	FN	496	196	9.4	10.9	79.4	2.4	M
10	FR	264	121	8.8	10.4	75.8	2.8	M
11	GH	43	29	6.7	8.6	79.0	2.1	C
12	HQ	351	245	10.2	11.9	75.7	3.1	B
13	HW	297	230	10.6	12.3	74.7	3.3	A
14	KT	131	8	9.3	10.8	75.9	2.8	B
15	MB	259	167	10.0	11.7	74.9	3.2	A
16	NA	138	73	9.7	11.4	76.2	2.9	A
17	PH	53	25	8.8	10.5	75.5	2.8	M
18	PK	101	46	10.5	12.2	75.5	3.2	C
19	RF	127	73	8.6	10.2	79.0	2.3	C
20	ST	689	187	10.6	12.2	75.0	3.2	B
21	SW	72	166	7.8	9.5	77.0	2.4	C
22	VS	10	43	7.7	9.2	79.7	2.1	A
23	WP	188	22	7.7	9.2	79.3	2.2	B
24	WR	102	46	8.9	10.6	76.5	2.7	M
25	WU	2	45	7.6	9.1	79.4	2.1	A
Mean		210	105	8.9	10.5	76.9	2.7	–

Sites specific observations of the number of *I. ricinus* nymphs N per 100 m^2^ and environmental variables from gridded measurements comprising temperature T in °C, relative humidity RH in %, saturation deficit SD in hPa as well as land cover classes A (agricultural land), B (broad-leaved forest), C (coniferous forest) and M (mixed forest) for 2013 and 2014.

The spatial resolution of the density map was selected to be 30 arc seconds, which corresponds to grid lengths of about 0.9 km (latitude) and 0.6 km (longitude). Climate variables described above have slightly lower resolutions and were disaggregated using the bi-linear interpolation of the R package *raster* [31, 32]. Categorical CORINE data, however, were provided with the higher resolution of 3 arc seconds. Therefore, they were aggregated to the 30 arc sec grid by selecting the most frequent land class within each grid box.

### Predictive statistical model

To predict the nymphal densities (N) in units of nymphs/100 m^2^, Poisson regression models were applied. The environmental variables height above sea level (H), temperature (T), relative humidity (RH), saturation deficit (SD) and the 4 land cover classes (LC) were selected as explanatory variables.

$$log_{e} \left( N \right) = \beta_{0} + \beta_{1} H + \beta_{2} T + \beta_{3} RH + \beta_{4} SD + \mathop \sum \limits_{i = 5}^{8} \beta_{i} LC_{i}$$

While the height and the climate variables were integrated in the model as numerical values, the land cover is considered as a categorical variable (classes A, B, C and M). For all other land cover classes the tick density is known to be very low or even zero. They comprise urban areas and water bodies for which no tick density was estimated. McFadden’s pseudo R_p_^2^ (the coefficient of determination R^2^, adapted for additional categorical variables such as LC) and the root mean square error (RMSE) were selected as goodness-of-fit measures. The final model was evaluated by leave-one-out cross-validation (LOOCV).

All analyses and graphical presentations were conducted using the open-source statistical computing environment R [31]. The gridded data were processed with the R-package raster [32].

## Results

**Fig. 3:**
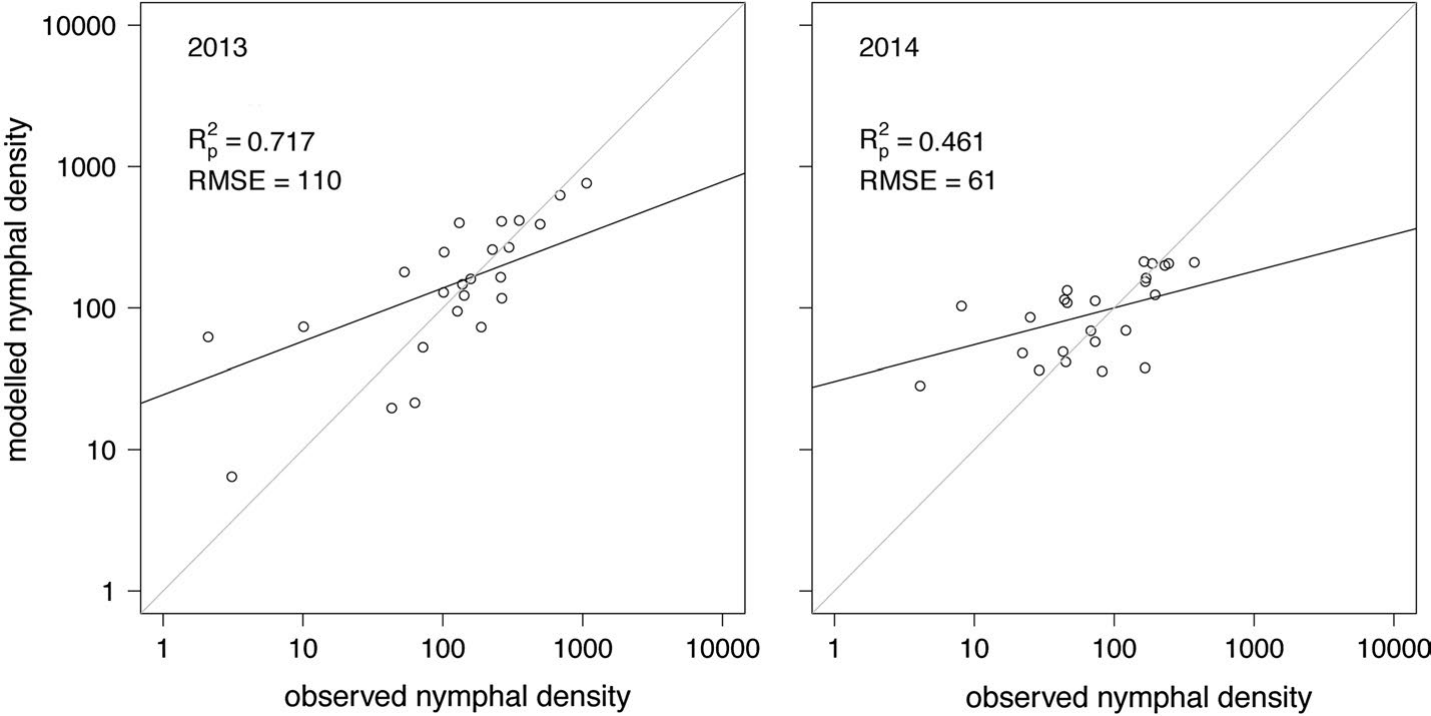
Observed vs. modelled *Ixodes ricinus* nymphs per 100 m^2^. Comparison of observed vs. modelled nymphal densities using gridded explanatory variables for 2013 (*left*) and 2014 (*right*). The model performance is expressed by explained pseudo variances R_p_^2^ and root mean square errors (RMSE).

The results of the field study were summarised in Table 2. For each of the 25 sampling locations described in Table 1, the total number of *I. ricinus* nymphs flagged monthly during 1 year is given together with the air temperatures for 2013 and 2014 and the long-term mean of the relative humidity, both extracted from gridded data provided by the German weather service (Deutscher Wetterdienst, DWD). Additionally, the calculated saturation deficit and land cover classifications are listed. Table 2 shows a wide range of values for the flagged nymphs. In 2013 for example, nymphal densities range from N = 0 at the mountain site FB (Feldberg) or N = 3 at AH (Allerheiligen) to N = 1,066 at BT (Botnang). Thus, for an optimal representation nymphal densities were plotted on a logarithmic scale. Solving the Poisson regression model to predict nymphal densities for 2013 and 2014 using the gridded explanatory variables results in the scatterplots depicted in Fig. 3. The Poisson regression model performs slightly better with the explorative variables of the year 2013. Looking at McFadden’s pseudo R_p_^2^, a total of 71.7 % of the variation in the observed nymphal density was explained by the modelled nymphal density in 2013. In contrast, only 46.1 % of the explained variance was estimated for 2014. The regression coefficients β for both models are summarised in Table 3. While for 2013 all of the explanatory variables contribute highly significantly to the performance of the model, for 2014 only three variables depict a significant contribution. Further, a collinearity of some variables was estimated. Although this collinearity does not influence the explained variances, it leads to regression parameters, which are not generally applicable (see the discussion in the next section).

**Table 3:** Summary of regression models for 2013 and 2014

	β	SE	z	p
Model for 2013				
Intercept	61.8768	3.3075	18.7080	<0.001***
H	0.0041	0.0003	12.9070	<0.001***
T_2013_	2.5372	0.0818	31.0230	<0.001***
RH	−0.8307	0.0377	−22.0370	<0.001***
SD	−6.5349	0.2913	−22.4320	<0.001***
Factor(LC) B	0.4321	0.0420	10.2880	<0.001***
Factor(LC) C	−0.5305	0.0675	−7.8600	<0.001***
Factor(LC) M	0.1773	0.0518	3.4260	<0.001***
Model for 2014				
Intercept	−0.7546	4.4926	−0.1680	0.867
H	0.0021	0.0004	5.3510	<0.001***
T_2014_	1.0116	0.1108	9.1280	<0.001***
RH	−0.0601	0.0521	−1.1520	0.249
SD	−0.6471	0.3934	−1.6450	0.100
Factor(LC) B	0.0791	0.0487	1.6260	0.104
Factor(LC) C	−0.3533	0.0731	−4.8340	<0.001***
Factor(LC) M	−0.0318	0.0681	−0.4660	0.641

For each explanatory variable the regression coefficient β, the standard error SE, the z-value (test statistics) and the *p* value (significance) are given. Note that land cover classifications A, B, C and M are categorical variables set to 0 (false) or 1 (true), from which class A was selected as default (β = 0).

**Fig. 4:**
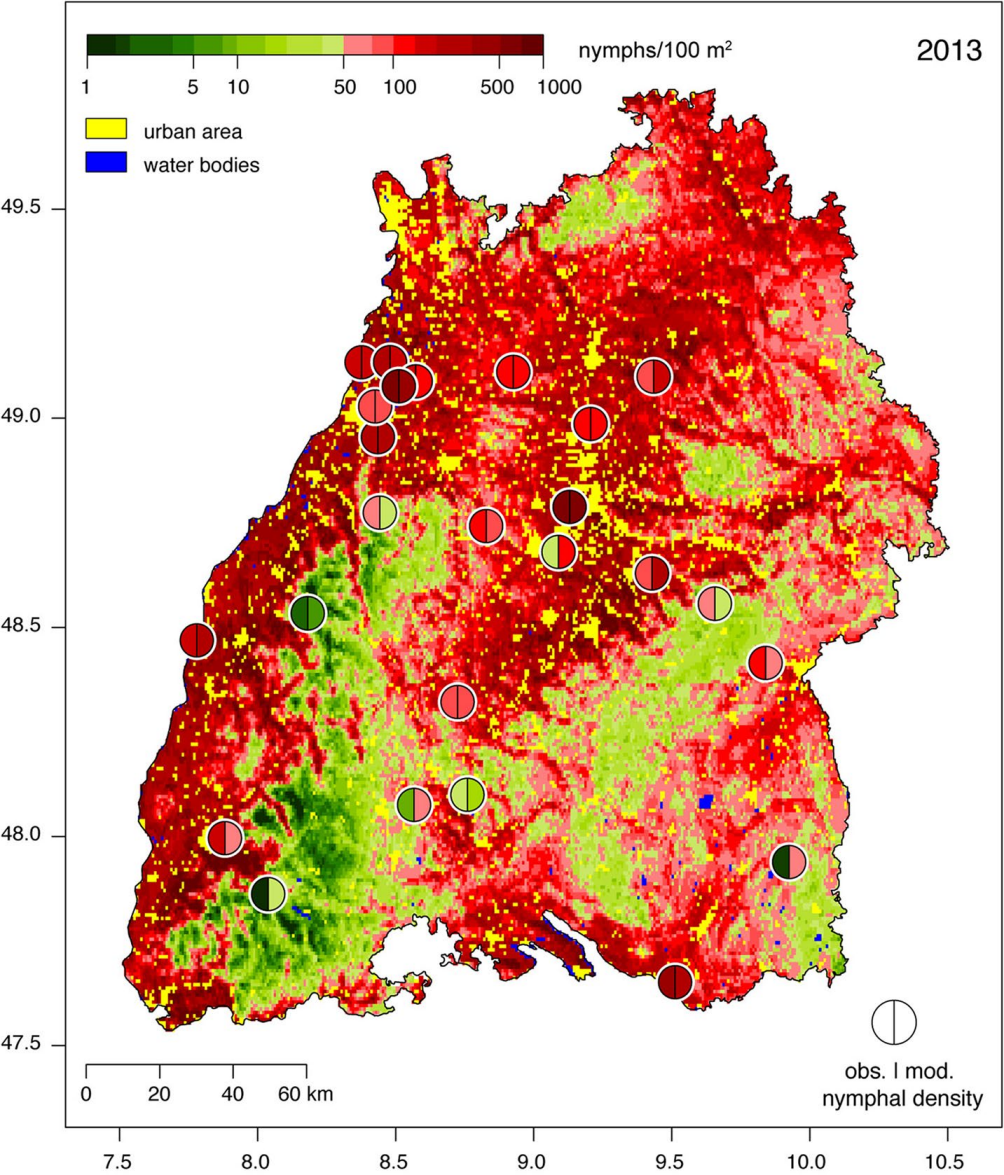
*Ixodes ricinus* nymphal ticks per 100 m^2^ for 2013. Map of the total number of nymphal ticks monthly flagged during 2013 and interpolated to the entire region of Baden-Württemberg, Germany. Sampling locations are marked by a *circle* showing both the observed (*left half*) and the modelled (*right half*) tick density.

**Fig. 5:**
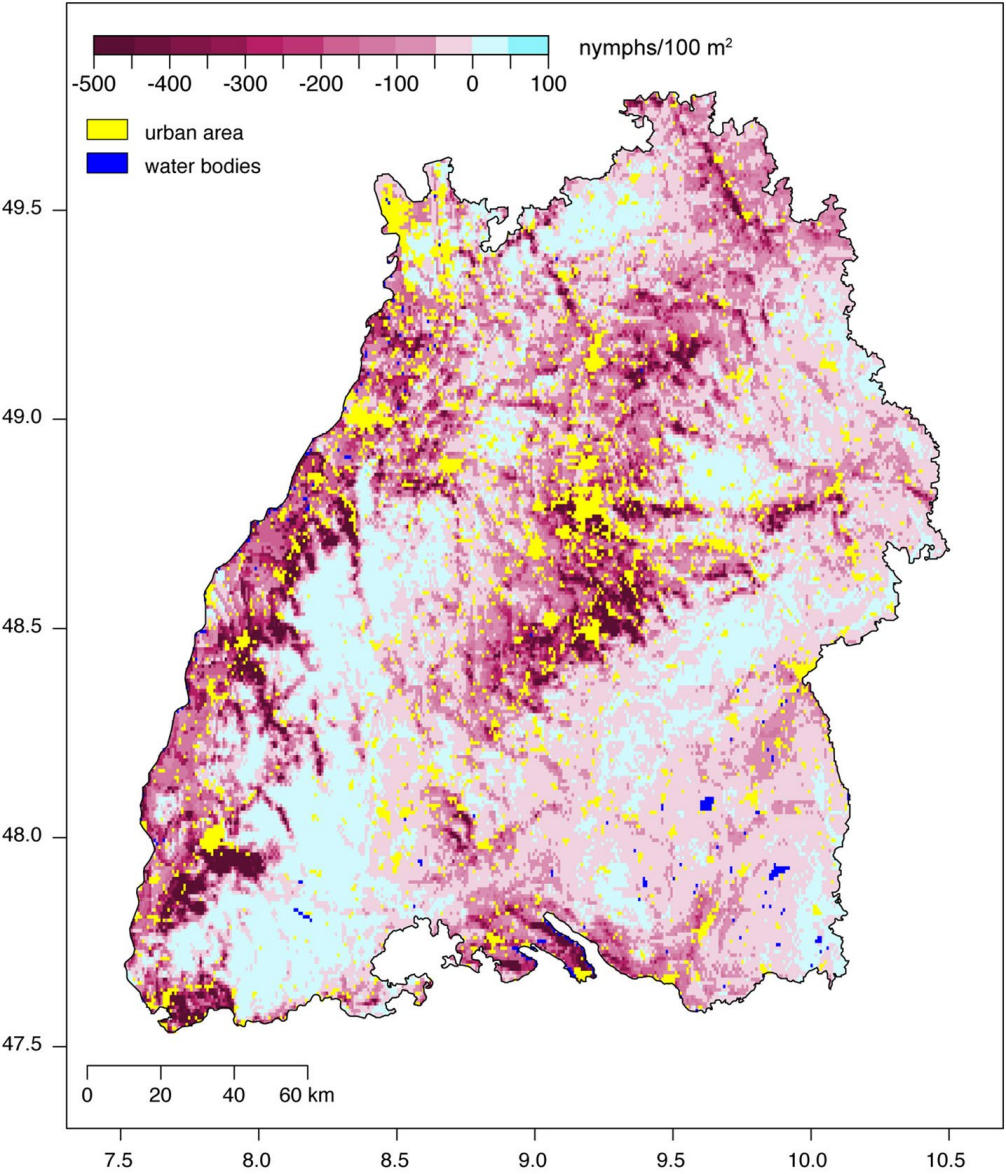
*Ixodes ricinus* nymphal ticks per 100 m^2^, difference 2014–2013. Map of the difference 2014–2013 of the total number of nymphal ticks monthly flagged in each year and interpolated to the entire region of Baden-Württemberg, Germany.

**Fig. 6:**
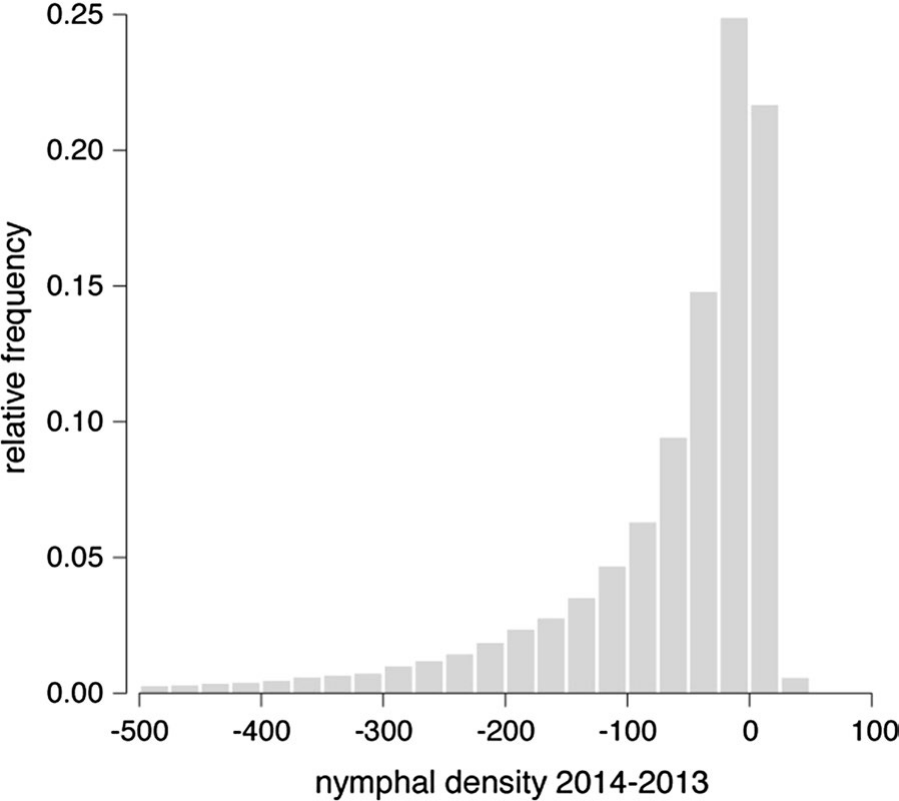
Frequency distribution of nymphal density, difference 2014–2013.

Poisson regression models with the parameters depicted in Table 2 were used to construct nymphal density maps of Baden-Württemberg for 2013 (Fig. 4) and 2014 (Additional file 1). Each map depicts the spatial distribution of the density of nymphs and should be interpreted as the amount of *I. ricinus* nymphs that may be collected by monthly flagging an area of 100 m^2^. Green grids represent regions with low nymphal densities (N = 0–50 nymphs/100 m^2^), while red gradations indicate regions with high nymphal densities (N = 50–1,000 nymphs/100 m^2^). Low densities of *I. ricinus* are restricted to higher altitudes in the Black Forest characterised by slightly fragmented coniferous forest habitats (Fig. 2c, class C). Moderate densities were estimated for all other hilly countryside with heights around 300–800 m characterised by shorter growing periods and/or forested areas with high proportions of coniferous forest. Very high densities were estimated for the warmest parts at altitudes below 400 m. This includes, in particular, the regions along the river Rhine and Neckar with surrounding areas, as well as the ambience of Lake Constance. The estimates for all other parts with warm to moderate climatic conditions tend to high nymphal densities. Urban areas and water bodies were excluded from the analysis (Fig. 4, yellow and blue areas). Comparing the maps for 2013 and 2014 depicts a similar distribution of the nymphal density, which is generally lower in 2014 (Fig. 5). These lower densities are caused by extraordinary high temperatures in 2014. The difference of the mean temperatures listed in Table 2 is 1.6 °C, which is of the same order as the temperature increase predicted by climatologists for the next 100 years. It seems that higher temperatures at lower altitudes are responsible for the observed decrease of the *I. ricinus* nymphal densities, while an increase of the generally lower temperatures at higher altitudes, e.g. in the black forest, cause an increase of the nymphal densities (Fig. 5). The frequency distribution of the difference between the nymphal densities mapped for 2013 and 2014 are depicted in Fig. 6. According to this frequency distribution about 50 % of the study region depicts only minor changes in the nymphal density (below ± 25 nymphs/100 m^2^). For the other 50 % of the area of Baden-Württemberg considerably lower nymphal densities were estimated for 2014.

To verify the Poisson regression model leave-one-out cross-validation (LOOCV) was applied [33]. It is a special case of the well-known p-out cross-validation with p = 1 as appropriate for the sampling size of n = 25 (flagging sites). LOOCV calculates all possible combinations of models calibrated with 24 sampling sites, which were validated with the remaining 1 site not used for calibration. Typically, LOOCV results were expressed by verification measures such as the prediction residual error sum of squares (PRESS) or the root mean square error (RMSE). For 2013, a value of RMSE = 227 nymphs/100 m^2^ was calculated providing a more realistic error measure than RMSE = 110 nymphs/100 m^2^ calculated with the whole set of sampling sites (Fig. 3). Due to generally lower observed nymphal densities, LOOCV results in lower errors of RMSE = 104 nymphs/100 m^2^ in 2014.

## Discussion

So far only few landscape scale tick density maps, such as those of the nymphal density of *I. pacificus* in California [6], were published. The *I. ricinus* maps presented here (Fig. 4, Additional file 1) contribute to the development of tick density maps compiled with statistical models. The nymphal densities are to be interpreted as the number of nymphs obtained by monthly flagging of an area of 100 m^2^. As a consequence of the flagging method only active questing ticks are sampled. Further, the proportion of questing ticks compared to the overall population depends on weather conditions [15]. Therefore, the questing tick densities are naturally lower than the actual densities [34, 35] and variable to a certain extend. This has to be taken into account by interpreting the results of the modelled nymphal densities. However, investigating only forests and forest-like habitats has a positive effect on the comparability of the results, since forest habitats provide comparable microclimatic conditions in contrast to other habitats such as meadows or clearances. Since forests offer very good survival conditions for *I. ricinus* ticks in Baden-Württemberg, modelled nymphal densities have to be interpreted as the upper end of the population density scale. Therefore, lesser population densities are to be expected in other habitats.

Uncertainties appeared in the modelling process, especially in the selection of explanatory variables. As suggested by experts [4], only biologically meaningful variables were considered instead of the frequently applied approach of reducing significant variables from a large set of possible explanatory variables. As a side effect, collinearities (e.g. between altitude and temperature) were estimated, which should be avoided in generally applicable models [4]. Due to the low number of explanatory variables used in the model, this is, however, not possible without reducing the model’s performance. Thus, the biological significance was higher valuated than statistical features and no generally applicable regression parameters were given. Instead, the regression parameters were separately fitted for each year and must be fitted again if further years will be investigated. In so doing, the model is demonstrably usable for mapping, i.e. spatial interpolation, but could not be used to predict future scenarios.

**Fig. 7:**
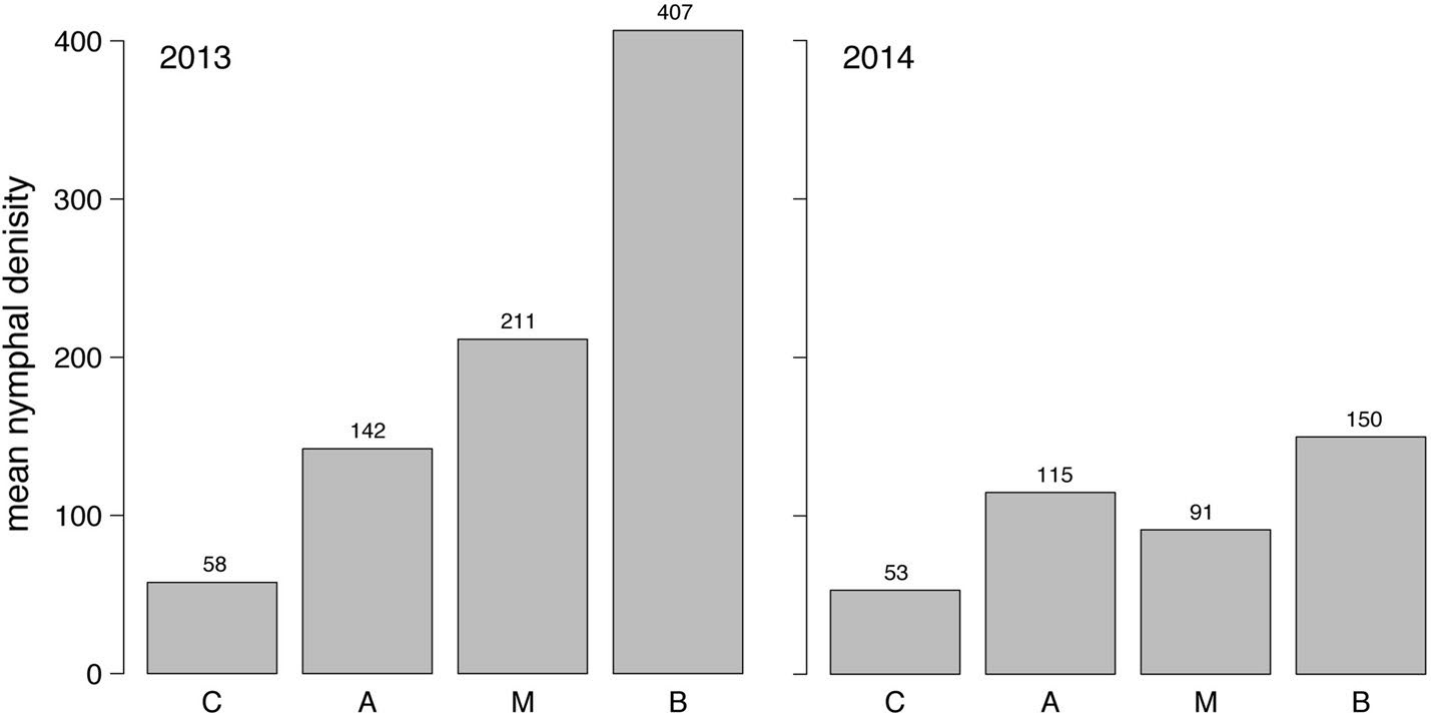
*Ixodes ricinus* nymphal densities for different land cover classes. Mean nymphal ticks per 100 m^2^ collected at sites classified as coniferous forest (*C*), agricultural area (*A*), mixed forest (*M*) and broad-leafed forest (*B*). While in 2013 nymphal densities of all land cover classes are significant different from those of the default class A (*left*), in 2014 only nymphal densities of C differs significantly from those of A (*right*). Lower nymphal densities in 2014 compared to 2013 are mainly related to lower densities in mixed (*M*) and broad-leafed forests (*B*).

By the choice of the generalized linear model (GLM) it was possible to include the categorical variable land cover. As a result, the performance of the statistical model increased and the tick maps could be calculated with the very high spatial resolution of 0.5 km^2^. The mean nymphal tick densities of the four land cover (LC) classes are depicted in Fig. 7. For the climatological unremarkable year 2013, a doubling rule may be derived from it. By the transition from one LC class to another, in the order of the classes C-A-M-B, the nymphal density of *I. ricinus* doubles. As expected, the statistically estimated density of *I. ricinus* is minimal in coniferous forests and maximal in broad-leafed forests. For the extraordinary hot year 2014, however, the nymphal densities of the LC classes M and B were not significantly different from class A. Only the nymphal densities in LC class C were significantly lower than in class A as depicted by the p-values of the regression coefficients in Table 2. In succession this might be responsible for the lower explained variance of the Poison regression model applied to the data of 2014. In this context it should be noted that A is the default LC in the Poisson model (as described above). Further, the definition of class A is notable, which contains arable land, permanent crops, pastures and heterogeneous agricultural areas. However, for a final statement concerning nymphal densities and LC longer time series of observations are needed. Although it is well known that *I. ricinus* is present even in city parks [36], no tick densities were estimated for urban areas. Tick habitats in urban areas were treated as sub-scale and not resolved by the maps presented here.

The model performance was expressed by explained variances of R_p_^2^ = 71.7 % for the model fitted with the data of 2013 and R_p_^2^ = 46.1 % for the model of 2014 (Fig. 3), indicating a high reliability of the *I. ricinus* density maps (Figs. 4, 5, Additional file 1). The results should be evaluated with regard to the uncertainties in observed tick densities as well as the low number of explanatory variables used for modelling. As a verification measure independent from observations used to calibrate the models, LOOCV errors of RMSE = 227 nymphs/100 m^2^ for 2013 and RMSE = 104 nymphs/100 m^2^ for 2014 were estimated. These errors are of the order of the mean nymphal density or 20 % of the maximum nymphal density, thus twice of the error estimated during the calibration process (RMSE = 110 nymphs/100 m^2^ for 2013 and RMSE = 61 nymphs/100 m^2^ for 2014). For high nymphal densities these errors are comparable to the observational error, i.e. the accuracy of flagging. It should be noted that it is also possible to fit the model with in situ measurements (Additional file 2), although gridded variables must be used to compile geographical maps.

Since the original research project was designed to estimate the density and distribution of ticks on the one hand, and to estimate the pathogen infestation on the other hand, the previous site selection had a focus on habitats and regions where ticks are likely to be found. Therefore, only a few sites with low and zero tick numbers were selected. This leads to uncertainties in observed and modelled nymphal densities, respectively. The inclusion of additional field data from land cover classes and altitudes with low tick densities would improve future model predictions. Varying abundance of several hosts, i.e. deer as the dominant host species for female ticks and small mammals as hosts for immature stages, are also likely to influence tick densities [34]. Future model improvements are expected by incorporating these data.

## Conclusions

Two years of *I. ricinus* observations from 25 sampling sites in the federal state Baden-Württemberg were used to develop a Poisson regression model. This predictive model was applied to compile very high-resolution maps depicting the spatial distribution of nymphal tick densities for the study area of 35,750 km^2^. The accuracy of the mapped tick densities was described by explained variances for the model calibration and prediction errors for the estimation of un-sampled sites. For the latter LOOCV was applied, indicating the reliability of the density maps. Although the methodology introduced here was shown to be suitable for compiling tick density maps, it may not be applied to other study regions without deriving new regression coefficients. For the same reason an application to predict climate change scenarios may not be recommended. Although field data were collected monthly (see the seasonal cycles in Additional file 3), only annually accumulated tick densities were considered to reduce the influence of observational errors and overdispersion [37], i.e. high nymphal densities due to local aggregation not resolved at the landscape scale (sub-grid scale effect).

Finally, the study is a first step towards a risk estimation for tick-borne diseases in Central Europe. Following the theory of the basic reproduction number for tick-borne diseases [38], this risk depends—beside other parameters—on the vector density or more exactly on the vector to host ratio. Mapping the risk of tick-borne diseases is therefore a challenge for the future. For other arthropod-borne diseases, however, vector density and disease risk maps are well established as described for Bluetongue [39] or Rift Valley Fever [40].

## Additional files

Additional file 1:
**Figure S1.** *Ixodes ricinus* nymphal ticks per 100 m^2^ for 2014. Map of the total number of nymphal ticks monthly flagged during 2014 and interpolated to the entire region of Baden-Württemberg, Germany. Sampling locations are marked by a circle showing both the observed (left half) and the modelled (right half) tick density. Additional file 2. Poisson regression model with in-situ measurements. **Table S1**. Environmental variables observed at the 25 sampling sites. Sites specific environmental variables from in-situ measurements comprising temperature T in °C, relative humidity RH in %, saturation deficit SD in hPa as well as land cover classes A (agricultural land), B (broad-leaved forest), C (coniferous forest) and M (mixed forest) for 2013 and 2014. **Table S2**. Summary of regression models for 2013 and 2014 using in-situ variables. For each explanatory variable the regression coefficient b, the standard error SE, the z-value (test statistics) and the p-value (significance) are given. Note that land cover classifications A, B, C and M are categorical variables set to 0 (false) or 1 (true), from which class A was selected as default (b = 0). **Figure S2**. Observed vs. modelled *Ixodes ricinus* nymphs per 100 m^2^. Comparison of observed vs. modelled nymphal densities using in-situ measured explanatory variables for 2013 (left) and 2014 (right). The model performance is expressed by explained pseudo variances R_p_^2^ and root mean square errors (RMSE). Additional file 3:
**Figure S3.** Time series of monthly Ixodes ricinus nymphal ticks per 100 m^2^. Selected sites for land classes C, A, M and B.
