# Supplementary material for: Estimating Ixodes ricinus densities on the landscape scale
Source: Int J Health Geogr. 2015 Aug 14;14:23. doi: 10.1186/s12942-015-0015-7 (PMC4536605; doi:10.1186/s12942-015-0015-7)
Supplement: Additional file 1: — Figure S1. Ixodes ricinus nymphal ticks per 100 m2 for 2014. Map of the total number of nymphal ticks monthly flagged during 2014 and interpolated to the entire region of Baden-Württemberg, Germany. Sampling locations are marked by a circle showing both the observed (left half) and the modelled (right half) tick density. [file 12942_2015_15_MOESM1_ESM.pdf]

Ixodes ricinus nymphal ticks for 2014

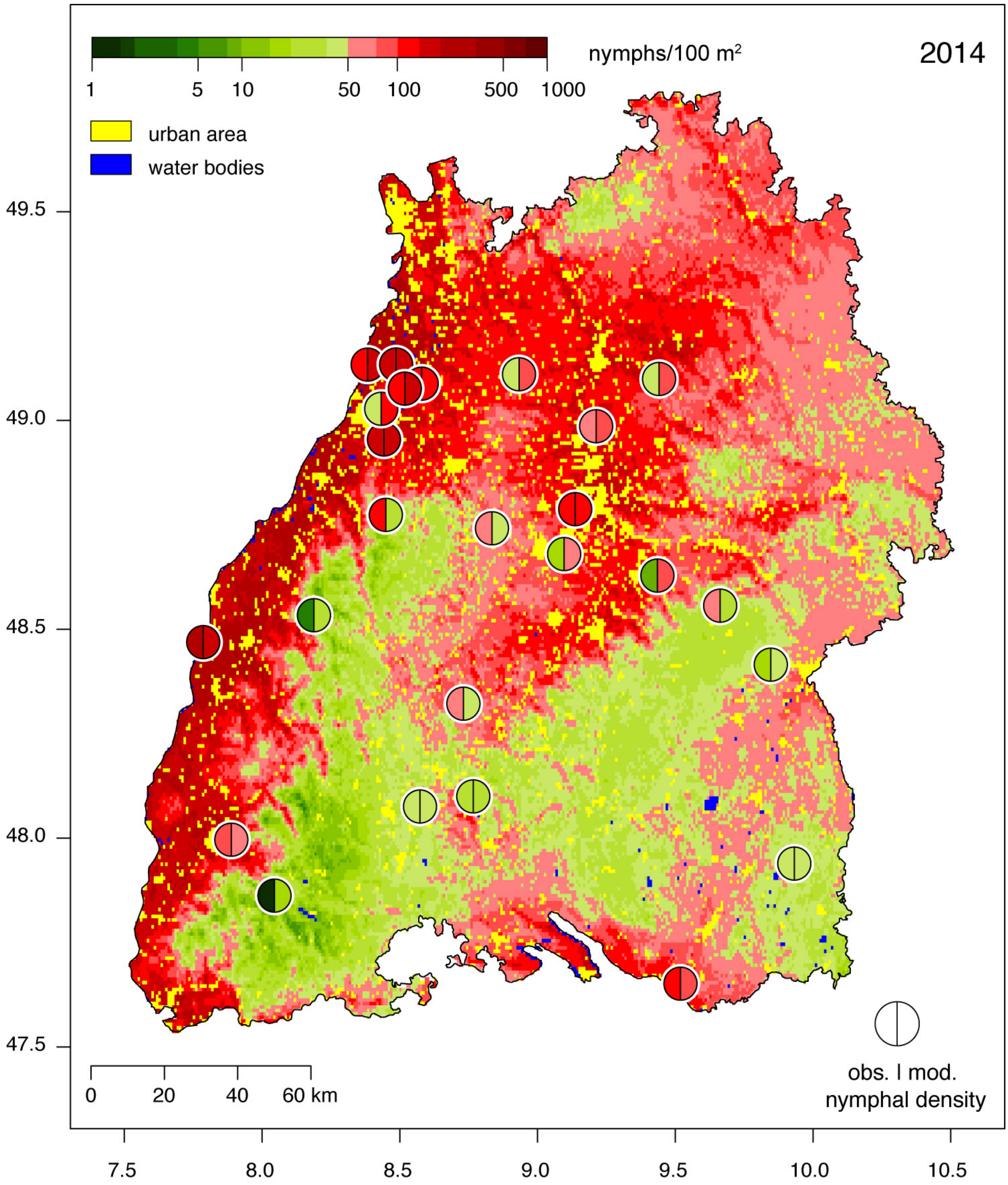

Figure S1 - *Ixodes ricinus* nymphal ticks per 100 m^2 for 2014.
Map of the total number of nymphal ticks monthly flagged during 2014 and interpolated to the entire region of Baden-Württemberg, Germany. Sampling locations are marked by a circle showing both the observed (left half) and the modelled (right half) tick density.
