# Supplementary material for: Estimating Ixodes ricinus densities on the landscape scale
Source: Int J Health Geogr. 2015 Aug 14;14:23. doi: 10.1186/s12942-015-0015-7 (PMC4536605; doi:10.1186/s12942-015-0015-7)
Supplement: Additional file 2. — Poisson regression model with in-situ measurements. Table S1. Environmental variables observed at the 25 sampling sites. Sites specific environmental variables from in-situ measurements comprising temperature T in °C, relative humidity RH in %, saturation deficit SD in hPa as well as land cover classes A (agricultural land), B (broad-leaved forest), C (coniferous forest) and M (mixed forest) for 2013 and 2014. Table S2. Summary of regression models for 2013 and 2014 using in-situ variables. For each explanatory variable the regression coefficient b, the standard error SE, the z-value (test statistics) and the p-value (significance) are given. Note that land cover classifications A, B, C and M are categorical variables set to 0 (false) or 1 (true), from which class A was selected as default (b = 0). Figure S2. Observed vs. modelled Ixodes ricinus nymphs per 100 m2. Comparison of observed vs. modelled nymphal densities using in-situ measured explanatory variables for 2013 (left) and 2014 (right). The model performance is expressed by explained pseudo variances Rp2 and root mean square errors (RMSE). [file 12942_2015_15_MOESM2_ESM.pdf]

Poisson regression model with in-situ measurements

Table S1 - Environmental variables observed at the 25 sampling sites.
Sites specific environmental variables from in-situ measurements comprising temperature T in °C, relative humidity RH in %, saturation deficit SD in hPa as well as land cover classes A (agricultural land), B (broad-leaved forest), C (coniferous forest) and M (mixed forest) for 2013 and 2014.

no	site	T_{2013}	T_{2014}	RH_{2013}	RH_{2014}	SD_{2013}	SD_{2014}	LC
1	AH	7.5	9.1	88.2	85.1	1.9	2.1	C
2	AL	10.2	11.7	88.3	88.3	1.8	1.9	B
3	AW	10.1	11.7	89.7	87.5	1.4	1.9	B
4	BT	9.1	10.6	89.8	87.7	1.6	1.8	M
5	CW	8.0	9.9	89.5	86.8	1.6	2.0	M
6	DS	7.1	8.9	86.5	85.9	1.9	1.9	B
7	EP	9.3	10.9	90.7	90.9	1.4	1.4	B
8	FB	4.4	5.8	94.9	90.5	0.7	1.1	C
9	FN	8.6	9.4	92.2	90.7	1.2	1.3	M
10	FR	9.9	11.5	84.8	85.3	2.4	2.4	B
11	GH	6.0	7.5	90.8	91.0	1.5	1.2	M
12	HQ	9.2	10.8	93.7	91.5	0.9	1.2	B
13	HW	10.1	11.8	88.3	84.6	1.8	2.5	M
14	KT	8.4	10.0	92.8	91.6	1.1	1.1	B
15	MB	9.8	11.4	84.7	84.1	2.4	2.5	B
16	NA	9.3	10.9	90.4	90.1	1.3	1.5	B
17	PH	8.0	9.2	90.3	90.8	1.2	1.2	M
18	PK	9.9	11.7	89.5	88.8	1.6	1.8	M
19	RF	8.0	10.1	90.6	90.0	1.4	1.5	A
20	ST	10.0	10.4	89.7	89.2	1.6	1.6	B
21	SW	8.4	9.8	84.1	81.3	2.5	2.7	M
22	VS	7.1	8.4	94.5	94.2	0.8	0.8	C
23	WP	7.4	9.5	89.2	87.6	1.7	1.9	B
24	WR	8.2	10.1	87.9	86.1	2.0	2.2	C
25	WU	7.1	8.5	90.6	90.1	1.3	1.3	A
mean		8.4	10.0	89.7	88.4	2.7	1.7	-

Table S2 - Summary of regression models for 2013 and 2014 using in-situ variables.

For each explanatory variable the regression coefficient β, the standard error SE, the z-value (test statistics) and the p-value (significance) are given. Note that land cover classifications A, B, C and M are categorical variables set to 0 (false) or 1 (true), from which class A was selected as default (β=0).

	**β**	**SE**	**z**	**p**	
**model for 2013**					
Intercept	-113.9000	4.5160	-25.2150	< 0.001	***
H	0.0028	0.0003	8.3490	< 0.001	***
T_{2013}	1.1150	0.0734	15.2000	< 0.001	***
RH_{2013}	1.1050	0.0456	24.2560	< 0.001	***
SD_{2013}	5.6930	0.2825	20.1520	< 0.001	***
factor(LC) B	0.2548	0.0962	2.6470	< 0.01	**
factor(LC) C	-1.4850	0.1325	-11.2060	< 0.001	***
factor(LC) M	0.5188	0.0955	5.4340	< 0.001	***
**model for 2014**					
Intercept	14.1022	4.9236	2.8640	< 0.01	**
H	-0.0026	0.0003	-10.3790	< 0.001	***
T_{2014}	-0.1489	0.0698	-2.1350	< 0.05	*
RH_{2013}	-0.0802	0.0535	-1.4970	0.134	
SD_{2013}	0.0779	0.3473	0.2240	0.823	
factor(LC) B	0.0901	0.1041	0.8660	0.387	
factor(LC) C	-0.7813	0.1420	-5.5030	< 0.001	***
factor(LC) M	0.0485	0.1054	0.4600	0.645	

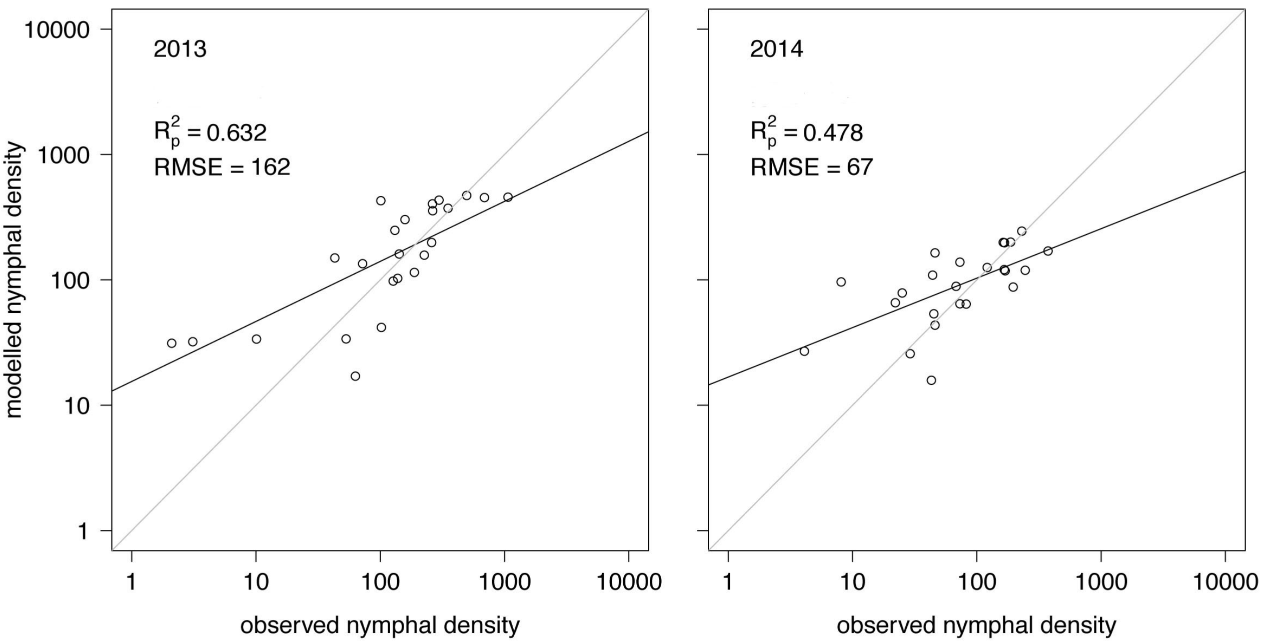

Figure S2 - Observed vs. modelled *Ixodes ricinus* nymphs per 100 m^2.
Comparison of observed vs. modelled nymphal densities using in-situ measured explanatory variables for 2013 (left) and 2014 (right). The model performance is expressed by explained pseudo variances R_p^2 and root mean square errors (RMSE).
