# Supplementary material for: Estimating Ixodes ricinus densities on the landscape scale
Source: Int J Health Geogr. 2015 Aug 14;14:23. doi: 10.1186/s12942-015-0015-7 (PMC4536605; doi:10.1186/s12942-015-0015-7)
Supplement: Additional file 3: — Figure S3. Time series of monthly Ixodes ricinus nymphal ticks per 100 m2. Selected sites for land classes C, A, M and B. [file 12942_2015_15_MOESM3_ESM.pdf]

Time series of *Ixodes ricinus* nymphal ticks

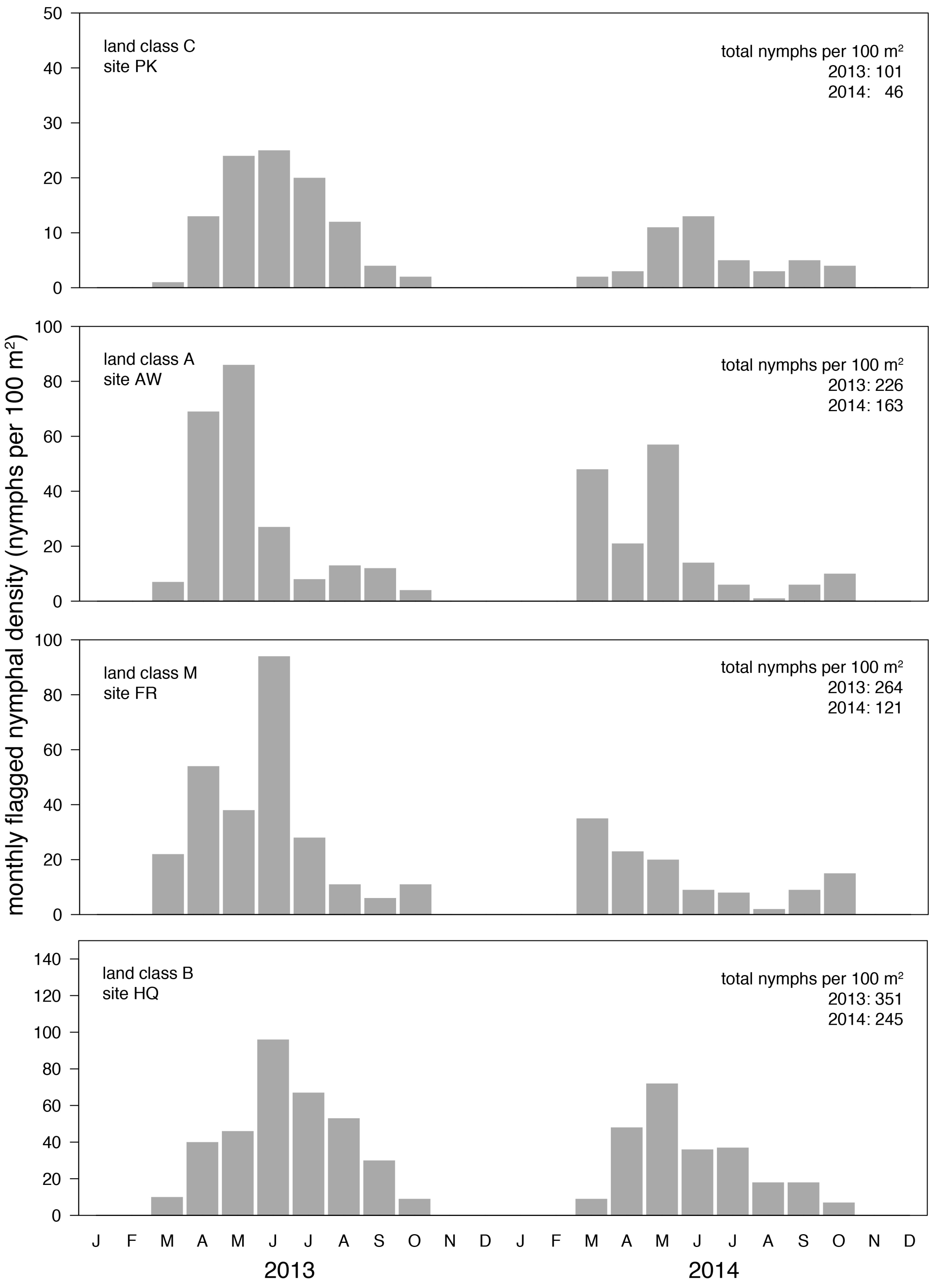

Figure S3 - Time series of monthly *Ixodes ricinus* nymphal ticks per 100 m^2.
Selected sites for land classes C, A, M and B.
